# Single-cell mass distributions reveal simple rules for achieving steady-state growth

**DOI:** 10.1128/mbio.01585-23

**Published:** 2023-09-06

**Authors:** Benjamin R. K. Roller, Cathrine Hellerschmied, Yanqi Wu, Teemu P. Miettinen, Annika L. Gomez, Scott R. Manalis, Martin F. Polz

**Affiliations:** 1 Division of Microbial Ecology, Centre for Microbiology and Environmental Systems Science, University of Vienna, Vienna, Austria; 2 Koch Institute for Integrative Cancer Research, Massachusetts Institute of Technology, Cambridge, Massachusetts, USA; 3 Department of Civil and Environmental Engineering, Massachusetts Institute of Technology, Cambridge, Massachusetts, USA; 4 Department of Biological Engineering, Massachusetts Institute of Technology, Cambridge, Massachusetts, USA; 5 Department of Mechanical Engineering, Massachusetts Institute of Technology, Cambridge, Massachusetts, USA; University of Hawaii at Manoa, Honolulu, Hawaii, USA

**Keywords:** physiology, cell size, *Vibrio*, single-cell methods, microfluidics

## Abstract

Optical density is a proxy of total biomass concentration and is commonly used for measuring the growth of bacterial cultures. However, there is a misconception that exponential optical density growth is equivalent to steady-state population growth. Many cells comprise a culture and individuals can differ from one another. Hallmarks of steady-state population growth are stable frequency distributions of cellular properties over time, something total biomass growth alone cannot quantify. Using single-cell mass sensors paired with optical density measurements, we explore when steady-state population growth prevails in typical batch cultures. We find the average cell mass of *Escherichia coli* and *Vibrio cyclitrophicus* growing in several media increases by 0.5–1 orders of magnitude within a few hours of inoculation, and that time-invariant mass distributions are only achieved for short periods when cultures are inoculated with low initial biomass concentrations from overnight cultures. These species achieve an effective steady-state after approximately 2.5–4 total biomass doublings in rich media, which can be decomposed to 1 doubling of cell number and 1.5–3 doublings of average cell mass. We also show that typical batch cultures in rich media depart steady-state early in their growth curves at low cell and biomass concentrations. Achieving steady-state population growth in batch culture is a delicate balancing act, so we provide general guidance for commonly used rich media. Quantifying single-cell mass outside of steady-state population growth is an important first step toward understanding how microbes grow in their natural context, where fluctuations pervade at the scale of individuals.

**IMPORTANCE:**

Microbiologists have watched clear liquid turn cloudy for over 100 years. While the cloudiness of a culture is proportional to its total biomass, growth rates from optical density measurements are challenging to interpret when cells change size. Many bacteria adjust their size at different steady-state growth rates, but also when shifting between starvation and growth. Optical density cannot disentangle how mass is distributed among cells. Here, we use single-cell mass measurements to demonstrate that a population of cells in batch culture achieves a stable mass distribution for only a short period of time. Achieving steady-state growth in rich medium requires low initial biomass concentrations and enough time for individual cell mass accumulation and cell number increase via cell division to balance out. Steady-state growth is important for reliable cell mass distributions and experimental reproducibility. We discuss how mass variation outside of steady-state can impact physiology, ecology, and evolution experiments.

## INTRODUCTION

Tracking microbial growth via optical density (OD) is fundamental and simple (1, 2), but it can also mislead by obscuring the processes underlying growth. A culture exhibiting exponential OD growth is often falsely equated with steady-state growth, despite ample literature stating otherwise (2 – 5). Bacterial cells alter their mass, external dimensions, and macromolecular composition as the nutrient availability and chemistry of the growth medium change in batch culture (4 – 8). The average cell in a batch culture only exhibits a consistent macromolecular composition when the population achieves steady-state growth. Steady-state population growth describes when the frequency distributions of all measurable cellular properties are time-invariant and it implies balanced growth, which is when all constituent parts of a cell are synthesized at the same exponential rate (3). OD is a proxy for the total mass concentration in a liquid medium (2, 9) and does not distinguish between changes in the cell number, average cell mass, or mass variation among cells. Therefore, OD measurements alone obscure key physiological transitions in batch culture, such as the entry into steady-state population growth.

Verifying steady-state population growth or balanced growth is laborious, but essential for examining cell size regulation (10, 11), cell cycle progression (12 – 14), and cell organization (15). Many independent aspects of growth must be measured for each culture while repeatedly performing dilutions to guarantee nutrients are available in excess of biosynthetic demand. In practice, many studies simply allow for a certain number of OD doublings (typically at least 10) in unchanging conditions to ensure cells are in an effective steady-state with a constant exponential growth rate in both OD and cell number (2, 16). Another practical way to verify if an effective steady-state has been achieved is to show the frequency distribution of any cell component does not change over time (2, 3). Cell mass is the output of all major biosynthetic processes and a constant mass distribution is a robust proxy for effective steady-state growth, though exceptions could exist if changes to growth, division, and death serendipitously balanced one another out. While achieving effective steady-state growth is undoubtedly important, less attention has been given to quantifying the coordination of cell mass and cell number dynamics during the transition into steady-state.

Here, we ask if simple guidelines can ensure bacterial cultures are reliably in effective steady-state growth and quantify how cells change as they transition into and out of this state. We grew two types of bacteria in commonly used rich, undefined growth media at several dilutions from a stationary phase parent culture: a typical laboratory bacterium [*Escherichia coli* K12 MG1655 in Lysogeny Broth (LB) at 37°C] and a recently isolated marine bacterium that has been minimally passaged in the laboratory (*Vibrio cyclitrophicus* 1G07 in Marine Broth 2216 at 25°C). We measured growth in batch culture via optical density, and also with a microfluidic mass sensor to capture single-cell buoyant mass and cell concentration (17, 18). We use the term mass in place of buoyant mass for clarity throughout the rest of this manuscript, unless specifically noted otherwise. Bacterial physiologists have debated if growth media made of complex and undefined nutrient mixtures, such as yeast extract or hydrolyzed protein, can support true steady-state growth (19) because the nutritional environment changes too rapidly, due to either sequential consumption of the multiple available carbon sources over time (5, 20, 21) or low divalent cation availability (19). Regardless of whether a perfect steady-state truly occurs, cultures in these undefined media do attain an effective steady-state growth at low cell concentrations in batch (5, 22) or with constant nutrient replenishment (23). Therefore, we investigated if the mass dynamics of *E. coli* growing in LB differed substantially from growth in defined, rich growth media designed for reproducible physiological experimentation (24) with two different primary carbon sources. While our measurements (mass distributions, OD, and cell number) can never rule out variation in some unmeasured biomass component (e.g., a particular protein), they do allow us to determine if there are general rules for achieving and departing from effective steady-state growth among diverse bacteria in commonly used cultivation conditions at different growth rates.

## RESULTS AND DISCUSSION

**Fig 1:**
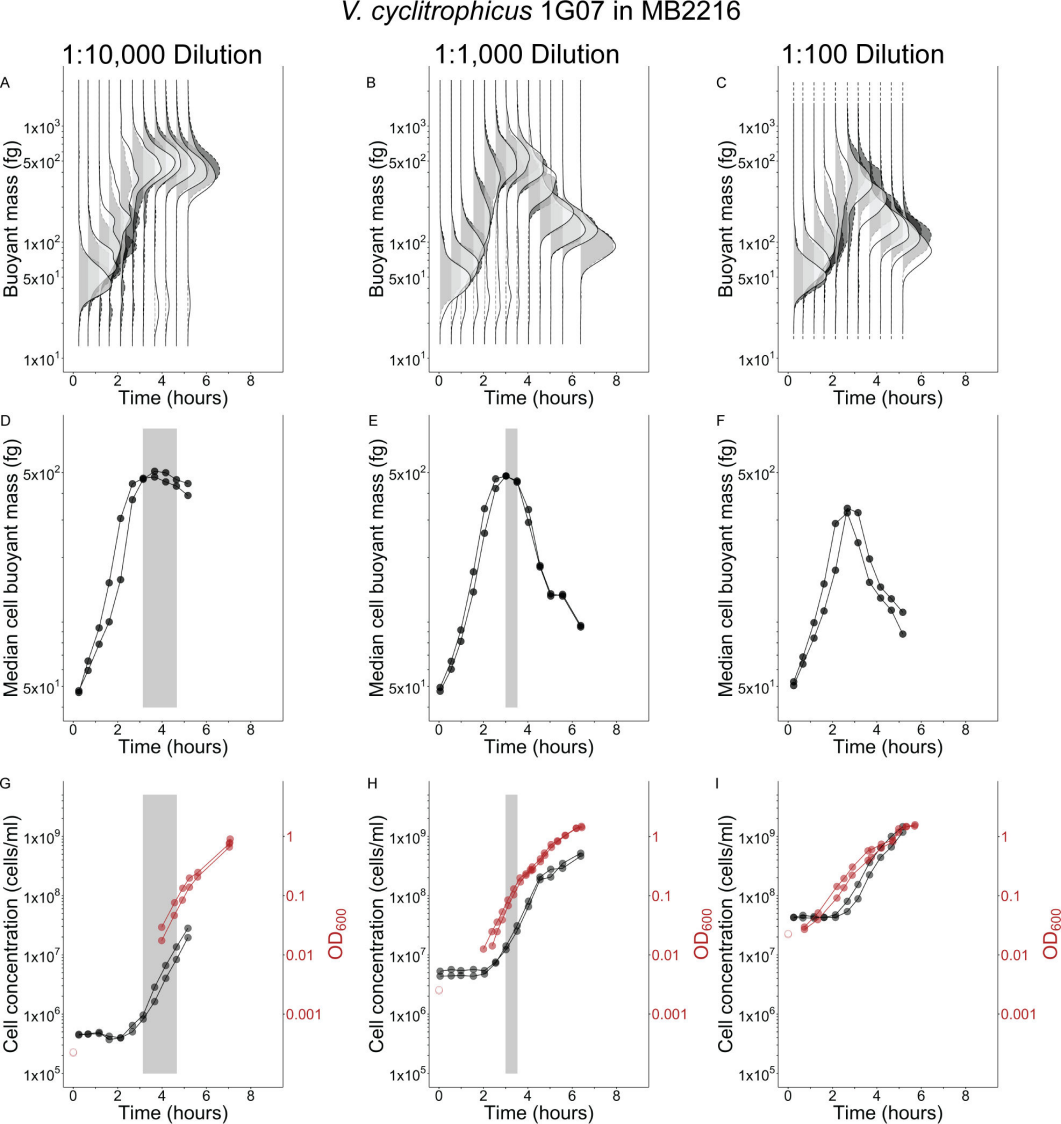
Effective steady-state growth of *V. cyclitrophicus* in Marine Broth 2216 (MB2216) batch cultures is short-lived and depends on inoculum concentration Buoyant mass distributions (**A–C**), median buoyant mass (**D–F**), cell concentration (**G–I**), and optical density 600 nm (OD_600_). (**G–I**) for two biological replicates [two shades of gray (A–C), two symbols (D–I)] of cultures inoculated into MB2216 medium with varying dilutions of a stationary phase culture [1:10,000 (A, D, G); 1:1,000 (B, E, H); 1:100 (C, F, I)]. Gray boxes (**D, E, G, H**) illustrate time periods when steady-state growth was effectively achieved (if ever). Solid lines drawn between points (**D–I**) connect consecutive observations for each replicate. Unfilled red symbols are provided as reference for the expected inoculum starting OD_600_ based on the measured OD_600_ of the stationary-phase parent culture at the time of inoculation and the known dilution factor, since the 1:1,000 and 1:10,000 dilutions brought the starting OD_600_ of experimental cultures below the lower limit of accuracy for the spectrophotometer (0.01 units OD_600_). Sample size of cells per replicate and time point provided in Table S1.

**Fig 2:**
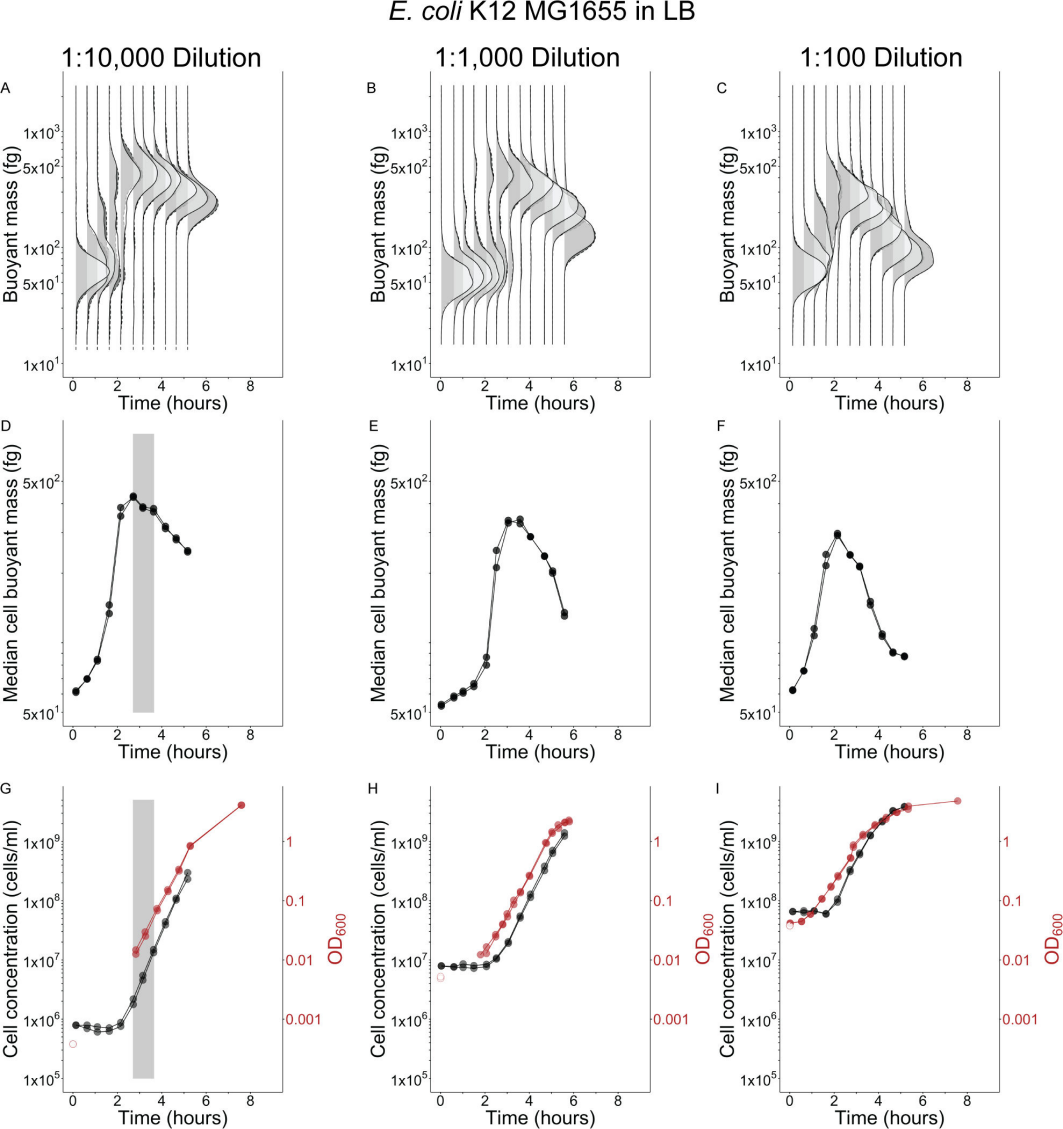
Effective steady-state growth of *E. coli* in Lysogeny Broth (LB) batch cultures is short-lived and depends on inoculum concentration Buoyant mass distributions (**A–C**), median buoyant mass (**D–F**), cell concentration (**G–I**), and optical density 600 nm (OD_600_) (**G–I**) for two biological replicates [two shades of gray (A–C), two symbols (D–I)] of cultures inoculated into LB medium with varying dilutions of a stationary phase culture [1:10,000 (A, D, G); 1:1,000 (B, E, H); 1:100 (C, F, I)]. Gray boxes (**D, G**) illustrate time periods when steady-state growth was effectively achieved (if ever). Solid lines drawn between points (**D–I**) connect consecutive observations for each replicate. Unfilled red symbols are provided as reference for the expected inoculum starting OD_600_ based on the measured OD_600_ of the stationary-phase parent culture at the time of inoculation and the known dilution factor, since the 1:1,000 and 1:10,000 dilutions brought the starting OD_600_ of experimental cultures below the lower limit of accuracy for the spectrophotometer (0.01 units OD_600_). Sample size of cells per replicate and time point provided in Table S1.

The ability of a batch culture to achieve steady-state growth depends on the initial OD, that is total mass concentration, in the culture. We performed longitudinal experiments of two species by making dilutions of either 1:100, 1:1,000, or 1:10,000 from overnight stationary phase cultures into commonly used undefined, rich media to vary the initial OD, then measured population dynamics over about 5 h. The median cell mass immediately increased for *E. coli* in LB and *V. cyclitrophicus* in MB2216 with no apparent lag time upon encountering fresh medium (Fig. 1A through F; Fig. 2A through F), while cell number did not increase for about 2.5 h (Fig. 1G through I; Fig. 2G through I). The immediate median cell mass increase is also reflected as rapid changes in OD (Fig. 1I and 2I). *V. cyclitrophicus* and *E. coli* achieve effective steady-state growth in the 1:10,000 dilution cultures because they exhibit three properties over a sustained period: time-invariant mass distributions, a constant median cell mass, and near-parallel increases in OD and cell number (effective steady-state highlighted by gray boxes in Fig. 1 and 2). While *V. cyclitrophicus* briefly achieved effective steady-state growth in the 1:1,000 dilution cultures (Fig. 1B, E, and H), *E. coli* did not (Fig. 2B, E, and H), and neither species achieved effective steady-state growth in the 1:100 dilution cultures (Fig. 1C, F, and I; Fig. 2C, F, and I) despite having obvious exponential increases in OD (Fig. 1I; Fig. 2I). The 1:100 cultures of both species exhibit a median cell mass decrease around the time the first doubling of cell concentration occurs (Fig. 1F and I; Fig. 2F and I), suggesting that single-cell and population growth are never coordinated in this dilution of longitudinal experiments. Large dilutions are necessary to reliably achieve effective steady-state growth.

**Fig 3:**
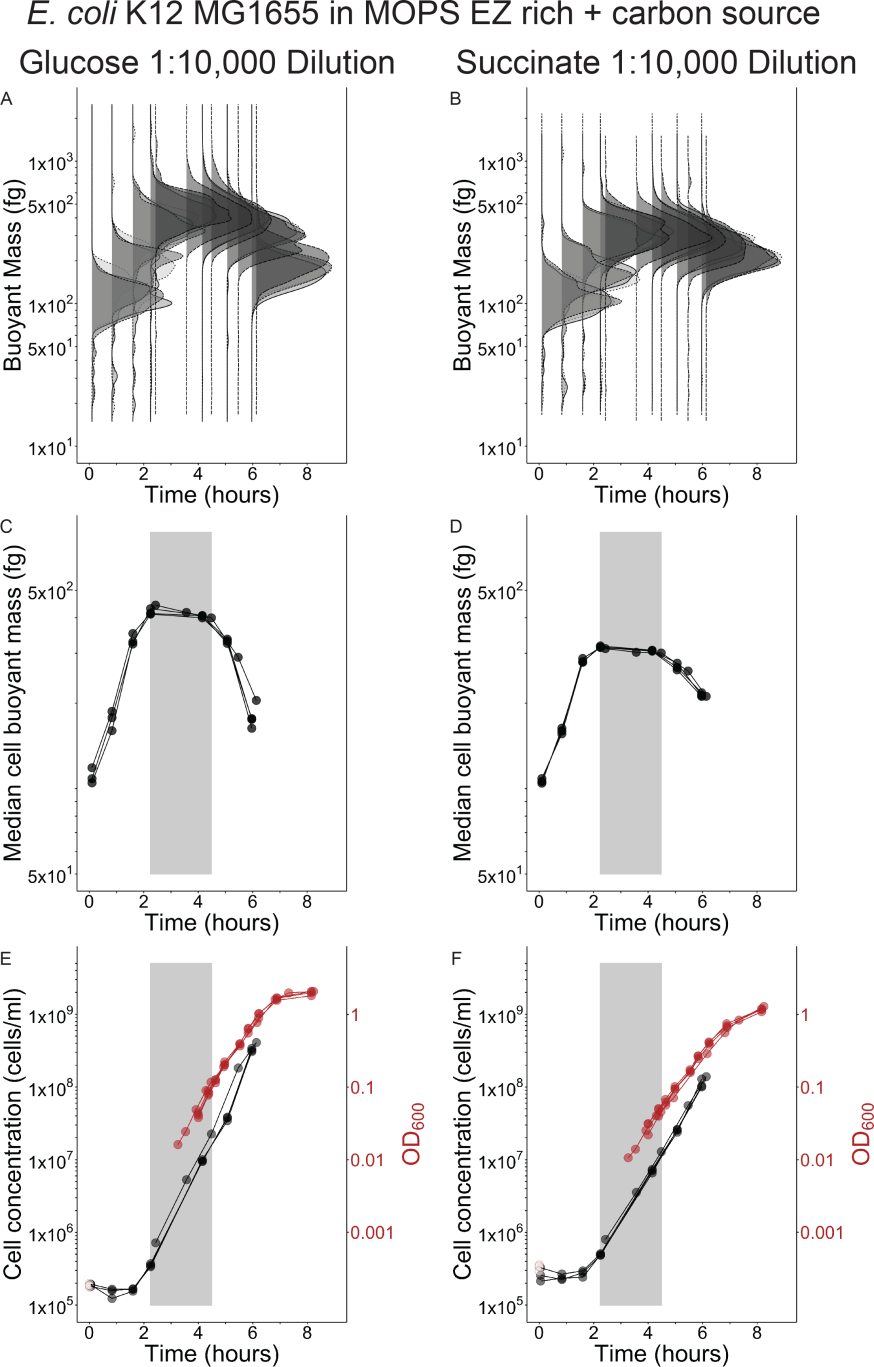
Effective steady-state growth of *E. coli* in chemically defined media supporting different growth rates is also short-lived buoyant mass distributions (**A and B**), median buoyant mass (**C and D**), cell concentration (**E and F**), and optical density 600 nm (OD_600_) (**E and F**) for four biological replicate [four shades of gray (A and B), four symbols (C–F)] cultures inoculated into defined medium with either glucose (**A, C, E**) or succinate (**B, D, F**) as a primary carbon source after 1:10,000 dilution from a stationary phase culture. Gray boxes (**C and F**) illustrate time periods when steady-state growth was effectively achieved and growth rates measured during this window (glucose = 1.73 ± 0.04 h^−1^ standard deviations using cell concentration and 1.80 ± 0.12 h^−1^ using OD, succinate = 1.38 ± 0.03 h^−1^ using cell concentration and 1.31 ± 0.16 h^−1^ using OD) match well to expected steady-state growth rates from the literature for *E. coli* in these media: glucose = 1.72 h^−1^, succinate = 1.31 h^−1^ (16). Solid lines drawn between points (**D–I**) connect consecutive observations for each replicate. Unfilled red symbols are provided as reference for the expected inoculum starting OD_600_ based on the measured OD_600_ of the stationary-phase parent culture at the time of inoculation and the known dilution factor, since the 1:10,000 dilutions brought the starting OD_600_ of experimental cultures below the lower limit of accuracy for the spectrophotometer (0.01 units OD_600_). Sample size of cells per replicate and time point provided in Table S1.

We then asked if a 1:10,000 dilution longitudinal experiment with *E. coli* in defined, rich media supporting different steady-state growth rates would produce similar cell mass dynamics to the LB medium at the same dilution. MOPS EZ rich medium produces reliable steady-state growth rates for *E. coli*, as well as the ability to modulate steady-state growth rate, with concomitant changes in average steady-state cell mass, when different primary carbon sources are provided (16). We found that a 1:10,000 dilution of *E. coli* allowed for effective steady-state growth in two different primary carbon sources (gray boxes in Fig. 3A, C, and E = MOPS EZ rich + 0.4% glucose or Fig. 3B, D, and F = MOPS EZ rich + 30 mm succinate). The effective steady-state median mass was larger in glucose, which permits faster growth than succinate (Fig. 3C and D). Growth rates during the effective steady-state growth period calculated from OD and cell concentration (Fig. 3E and F, gray boxes) matched previously reported steady-state growth rates for these media (16), further supporting our approach for classifying steady-state growth in longitudinal experiments. While the initial median mass of *E. coli* in MOPS EZ rich media at inoculation is larger (Fig. 3C and D) than for LB (Fig. 2D through F), the median mass dynamics of *E. coli* do not differ substantially between any medium.

The number of OD, or total mass, doublings required for reaching steady-state in longitudinal experiments was fewer than we assumed based on typical literature guidelines of 10 doublings (2, 16). At the time effective steady-state begins in LB and MB2216 longitudinal experiments, the median cell had undergone about three median mass doublings (*V. cyclitrophicus:* 53–492 fg, *E. coli*: 63–420 fg), while the cell concentration had doubled (Fig. 1G and H; Fig. 2G) combining to four total mass doublings. This number is slightly smaller in MOPS EZ rich media, with three total mass doublings at the time of effective steady-state in MOPS EZ rich + glucose (1 cell concentration doubling + 2 median mass doublings: 110–425 fg) and 2.5 total mass doublings in MOPS EZ rich + succinate (1 cell concentration doubling + 1.5 median mass doublings: 106–315 fg). This total mass increase agrees well with the increase in OD from inoculation until steady-state onset for *V. cyclitrophicus* in MB2216 and *E. coli* in LB (Fig. 1H; Fig. 2G) but could not be checked in MOPS EZ rich media since the OD when steady-state growth began is below the lower limit of detection of our spectrophotometer. We have shown the number of total mass doublings necessary for ensuring steady-state growth can be as low as 2.5–4 when diluting a stationary phase culture into the same medium. Other single-cell studies examining different transitions between steady-states found it takes three to eight doublings to establish the new steady-state after a growth medium shift (25, 26), but they do not try to examine if their findings can be translated to traditional batch culture and optical density growth. Taken together, this suggests the guideline of 10 doublings for ensuring steady-state establishment is robust, but may be overly cautious.

**Fig 4:**
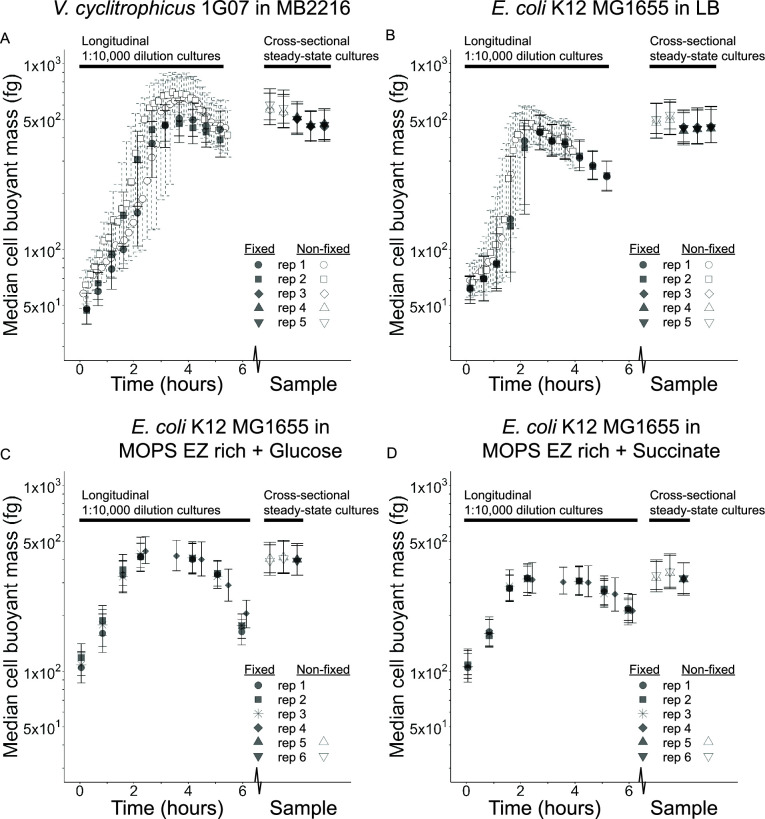
Simple rules for steady-state growth reliably generate cultures with consistent mass and the effect of chemical fixation on buoyant mass is generally small. Median buoyant mass (symbols for replicate cultures) and interquartile range (error bars) of fixed (black filled) or non-fixed (white-filled) samples from for either 1:10,000 dilution longitudinal cultures or cross-sectional steady-state cultures of all species and medium combinations [(A) *V. cyclitrophicus* 1G07 in MB2216, (B) *E. coli* K12 MG1655 in LB, (C) *E. coli* K12 MG1655 in MOPS EZ rich + glucose medium, (D) *E. coli* K12 MG1655 in MOPS EZ rich + succinate medium]. Fixed and non-fixed samples from the 1:10,000 longitudinal cultures in undefined media (**A and B**) are paired sub-samples from the same parent culture (*N* = 2, different shapes) grown on the same day, with fixed samples measured on a later date. Only fixed samples for 1:10,000 longitudinal cultures in defined media (*N* = 4, different shapes) are reported (**C and D**). Fixed and non-fixed samples from cross-sectional steady-state cultures have replicate cultures (*N* = 3 for panels (A and B), *N* = 2 for panels (C and D), different shapes), plotted on the *x*-axis within a given Sample. The different samples on the *x*-axis are treated as technical replicates and are as follows: The non-fixed cross-sectional samples represent two time points (before and after, always in that order on the *x*-axis) bracketing the time of fixation in all panels; The fixed cross-sectional samples (if multiple exist) were all taken at the same time point for a given panel, but represent a measurement after different durations of fixation [(A) 1 h, 1 day, and 6 days; (B) 1 h, 3 days, and 11 days; (C) 1 h; (D) 1 h, always in ascending order of time on the *x*-axis]. *P* values and effect sizes for steady-state fixed versus non-fixed samples in a mixed-effects ANOVA model (details of the model in methods) are as follows. Panel (A): 17.8% mass loss in fixed samples relative to non-fixed samples, *P* = 0.0225. Panel (B): 10.8% mass loss in fixed samples relative to non-fixed samples, *P* < 0.008. Panel (C): 2.8% mass loss in fixed samples relative to non-fixed samples, *P* = 0.434. Panel (D): 6.4% mass loss in fixed samples relative to non-fixed samples, *P* = 0.439. Sample size of cells per replicate and time point provided in Table S1.

The amount of mass gained by cells is enormous as they transition into effective steady-state growth following stationary phase in longitudinal experiments. While the median mass change we observed within any strain for a given medium could be influenced by changes to the composition of cellular dry mass via altered dry density, this effect is relatively small for *E. coli* in similar conditions (17). Therefore, *E. coli* buoyant mass in LB can be converted to dry mass using a multiplicative conversion factor between 3.1 (in stationary phase) to 3.6 (near steady-state) (17). *E. coli* alters its dry mass more in the first 3 h of our LB 1:10,000 dilution experiment (164–1,512 fg), than it does across 20 different growth media supporting steady-state growth rates between 0.31 and 1.72 per hour (240–1,180 fg) (16). Comparing buoyant mass among different media is complicated because the different media used in our study have different fluid densities, and different species likely have different dry densities (18). We therefore made conversion factors for addressing the impact of fluid density on the buoyant mass of polystyrene beads, but we caution that these do not apply directly to our bacteria because their dry densities are typically much larger than the beads (17, 18), so would be less affected by the difference in fluid density. MB2216 has the highest fluid density compared to other media (MB2216 to LB bead conversion factor = 1.6, Fig. S1), while all other media are quite similar in fluid density to one another (LB to MOPS EZ rich conversion factors = 0.941–0.974, Fig. S1). The chemical fixation of cells could also plausibly impact buoyant mass (27), so we assessed the impact of fixation over the duration of longitudinal experiments using paired sub-cultures for some media. We also compared fixed versus non-fixed samples for all strain/media combinations by generating independent steady-state experiments (using our simple rules for steady-state growth outlined below), which we term cross-sectional experiments. The impact of fixation in the longitudinal experiments was much smaller than the changes in mass over time (300–1,000% mass increase from initial mass to steady-state mass), but the dynamics of this experiment made statistical comparisons challenging (Fig. 4A and B). We quantified the effect size of fixation in cross-sectional experiments and found it was small in all strain/media combinations (2.8–17.8% mass loss in fixed samples) and only statistically significant for the undefined, rich media (Fig. 4A through D). However, the effect size of fixation did vary slightly depending on the strain and medium combination in cross-sectional experiments (Fig. 4A through D) and could depend on many organism/medium-specific differences among strains, such as differences in loss of diffusible metabolites during fixation. Experimentalists comparing mass in multiple conditions (e.g., different media) and species must account for within condition variation by establishing steady-state growth, as well as differences in medium fluid density, biomass composition, and sample preparation among species. The mass range clonal isolates can realize is vast, but cell mass is systematically responsive to the transient and long-term (16) nutrient conditions bacteria experience.

The exit from effective steady-state occurs very early in the longitudinal experiments at low OD and cell concentration in all media types. This has been reported previously for *E. coli* in LB (4, 5), and we observe effective steady-state exit to occur at an even lower OD and cell concentration in LB (Fig. 2G: ~0.1 units OD_600_ and ~1.5 × 10^7^ cells/mL). This early exit phenomenon seems to be generally true in rich media, since it is oberved for *E. coli* in both MOPS EZ rich media (Fig. 3E and F: ~0.1 units OD_600_ and 1–2 × 10^7^ cells/mL), and *V. cyclitrophicus* also exits at low OD and cell concentration (Fig. 1G and H: ~0.1 units OD_600_ and 2.5 × 10^7^ cells/mL). All OD growth curves also have subtle bends just after effective steady-state departure (Fig. 1G and H; Fig. 2G; Fig. 3E and F). While the absolute exit values for OD and cell concentration are similar to one another for the two species, the final yield of these species in their respective media differs (Final yield: *E. coli* in LB = ~5 units OD_600_ and ~7–8 × 10^9^ cells/mL; *E. coli* in MOPS EZ-rich glucose = ~2 units OD_600_ and ~2 × 10^9^ cells/mL; *E. coli* in MOPS EZ-rich succinate = ~3 units OD_600_ and ~3 × 10^9^ cells/mL; *V. cyclitrophicus* = ~2 units OD_600_ and ~5 × 10^9^ cells/mL). We also note that effective steady-state growth is only achieved if the concentration of cells at inoculation is at least one doubling below the cell concentration at which the population exits steady-state. Sequential depletion of preferred amino acids in rich media (5, 21) could be one possible explanation for the generic departure from effective steady-state growth at low cell densities we observe in longitudinal experiments, but demonstrating this would require experimentation beyond the scope of this work.

Although population averages are of central importance to steady-state phenomena, the mass distribution itself and how it shifts in these longitudinal experiments can provide additional insights into cell individuality in fluctuating conditions. The variability of cells from both species in undefined, rich media appears much larger than the variability of *E. coli* cells in defined, rich media (Fig. 1A through C; Fig. 2A through C; Fig. 3A and B; Fig. S2 through 4). Mass initially appears to have a log-normal distribution upon inoculation in all media and remains close to a log-normal distribution for *E. coli* in defined, rich media whether in effective steady-state or not (no change in robust coefficient of variation, Fig. S4A and B). In contrast, the distribution of both species in undefined, rich media quickly broadens after inoculation (increased robust coefficient of variation, Fig. S2A through C; S3A through C,) while the central tendency rapidly shifts upwards (Fig. 1A through C; Fig. 2A through C). This indicates that some cells add mass more quickly than others in undefined, rich media before effective steady-state growth is achieved. There is also no obvious sub-population of non-responsive cells in any media since the mass distribution at its maximum (2–3 h) is entirely larger than the initial mass distribution (Fig. 1A through C; Fig. 2A through C; Fig. 3A and B), though they may be too rare to detect with the number of cells we observed. Upon entry into effective steady-state growth, the mass distribution of both species in undefined, rich media returns to a more narrow log-normal distribution (Fig. 1A through C; Fig. 2A) with a smaller rCV (Fig. S2A through C; S3A through C), which is the expected distribution for cultures growing in steady-state growth (28). When both species exit effective steady-state growth in all media, the center of the distributions decreases but does not broaden (Fig. 1A through C; Fig. 2A through C; Fig. 3A and B) or have a change in rCV (Fig. S2A through C; S3A through C; S4A through B) indicating no change in variation among cells as they reductively divide to make offspring with ever decreasing size.

Microbiologists have known for a century that cell properties in liquid batch cultures are dynamic (6), but exactly how much cells alter their mass over time has been challenging to measure directly. We believe this information gap, along with misconceptions about OD, have led to the common misunderstanding that an exponential OD increase is equivalent to steady-state growth. While cultivation protocols to ensure steady-state growth are used by many microbial physiologists, adoption of these best practices is not universal or common knowledge across all microbiological disciplines. Here, we demonstrate that cell mass changes rapidly and substantially in batch culture, so care must be taken to achieve steady-state growth. While the exact amount of mass added upon dilution into fresh medium will depend on the specific strain and medium combination, it is a very large increase for the strains we grew in commonly used rich media.

We recommend that microbiologists intending to work with steady-state populations in batch culture on rich media perform the following steps.

1. Inoculate with a minimum of a 10,000-fold dilution from an overnight parent culture for *E. coli*-like cell yields (~10^9^ cells/mL).
2. Allow for at least four OD, or total mass, doublings (16-fold) prior to initiating an experiment.
3. Finish the experiment at 0.1 units OD_600_ and before the earliest bend in the growth curve.

Following these simple rules in independent cross-sectional experiments, we successfully generated cultures with median cell masses which are consistent with the median mass maxima we observed in the 1:10,000 dilution longitudinal experiments during effective steady-state growth (Fig. 4). While we sought to make our recommended rules simple, one major caveat with them is that OD measurements are never exactly comparable across devices or with different growth media and strain combinations (9). We believe our guidelines are cautious minimum standards and illustrate the complex interplay between individual and population growth all bacteria face in batch culture. We recognize that these criteria require an experimental decision about observing OD changes. This decision arises because the lower accuracy limit of OD_600_ in many spectrophotometers is near 0.01 (29), and starting from this point does not allow for four mass doublings, and therefore entry to steady-state, before the OD_600_ threshold of 0.1 is reached. One can either start from the OD_600_ of 0.01 and perform repeated dilutions of the culture until reaching four mass doublings, or start from outside of your observation window by performing a larger dilution. When performing a larger dilution, using the known OD_600_ of the parent culture and the dilution factor can reliably measure starting OD and ensure at least four total mass doublings finish in the range of 0.01–0.1 OD_600_.

The mass dynamics we documented could impact many experiments using batch cultivation, from comparing gene or protein expression to interpreting the dynamics of ecology and evolution experiments. For example, a common experimental design for studying microbial evolution is the serial daily passage of a batch culture with a 1:100 dilution (30). These experiments frequently observe strong selection on cell size and growth rate, and the rapid mass increase upon inoculation into fresh medium we observe is likely a daily occurrence in such evolution experiments (31, 32). Those cells which increase in mass fastest and grow fastest will have a large advantage compared to slow-responding cells. Serial passage experimental designs have also been used to study ecological dynamics within multispecies microbial communities (33, 34). All serial passage experiments likely face similar selective pressures on cell size and growth rate, while individual species may exhibit different mass accumulation responses. The amount of phenotypic heterogeneity in mass accumulation among clonal cells will also likely be impacted by the chosen strain and growth medium combination. Finally, based on final yield and our measurements, we see that the majority of total cell divisions in all of our longitudinal experiments occur outside of steady-state. The physiology of mass accumulation in batch culture is a complex phenomenon in clonal cultures, and could play a large role in the outcome of ecology and evolution experiments when a diversity of cell types is present in the culture.

## MATERIALS AND METHODS

### Bacterial strains and culture conditions

*E. coli* K12 MG1655 containing the pSIM6 plasmid (35) was provided by the laboratory of Dr. Robert Britton and grown in autoclaved and 0.1 µm filtered LB (Lennox) liquid medium (Carl Roth GmbH) or MOPS EZ rich liquid medium with either 0.4% glucose or 30 mM sodium succinate as a primary carbon source (24) (Teknova). *V. cyclitrophicus* 1G07 was isolated from cryopreserved seawater samples as previously described (36) and grown in boiled and 0.1 µm filtered Marine Broth 2216 (BD Difco) liquid medium (Fisher Scientific GmbH). *E. coli* was grown in pre-warmed media at 37°C shaking at 200 rpm, while *V. cyclitrophicus* was grown at 25°C shaking at 250 rpm.

The following cultivation protocol was followed in each longitudinal experiment. For each species, either two biological replicate cultures of undefined, rich media (LB or MB2216) or four biological replicate cultures of defined, rich media (MOPS EZ rich + glucose or MOPS EZ rich + succinate) were inoculated on day 1 from the same −80°C freezer stock into separate recovery cultures (5 mL medium in 13 mL test tube) and allowed to grow overnight. We define biological replicate as an independent culture originating from the same clonal freezer stock, but which was inoculated into a separate recovery culture and maintained separately for all further transfers. On the morning of day 2, the recovery cultures had all achieved stationary phase and high optical density, so they were inoculated with a 1:100 dilution into pre-cultures (50 µL into 5 mL medium in 13 mL test tube) and allowed to grow for an additional day. This ensured the cultures had several generations of growth to recover from cryopreservation and were also in stationary phase in the same medium used for experimentation for about 18 h. Longitudinal experimental cultures were inoculated on day 3, but inoculation protocol differed slightly between media types. For undefined, rich media several different dilutions (either 1:100, 1:1,000, or 1:10,000) of stationary phase pre-cultures were made into 30 mL of pre-warmed media. The 1:100 and 1:10,000 dilutions were performed on the same day and with the same two biological replicate pre-cultures as inoculant sources. For defined, rich media only 1:10,000 dilution experiments were performed and they were grown in 5–7 mL of pre-warmed media using four biological replicate pre-cultures as inoculant sources. All 1:10,000 experimental cultures were inoculated serially, first diluting the pre-cultures to generate a 1:100 culture, either a 30-mL experimental culture in undefined, rich media (0.3 mL pre-culture into 29.7 mL medium in 500 mL side-arm flasks) or a 1-mL transfer culture for defined, rich media (10 µL pre-culture into 990 µL medium in microcentrifuge tube). These 1:100 cultures were diluted further to generate the 1:10,000 experimental cultures for undefined, rich media (0.3 mL of 1:100 culture into 29.7 mL medium in 500 mL side-arm flasks) or defined, rich media (0.05–0.07 mL of 1:100 culture into 4.95–6.93 mL medium in 13 mL test tube). Experiments with the 1:1,000 experimental cultures were performed several months later with the same procedure until the inoculation of experimental cultures, where the replicate pre-cultures were diluted to generate the 1:1,000 experimental cultures (30 µL pre-culture into 30 mL medium in 500 mL side-arm flasks).

Experiments to compare fixed and non-fixed cells and to generate cross-sectional steady-state cultures following our simple rules (Fig. 4) were performed with the same cultivation protocol as the above 1:10,000 dilution cultures for all media, but had the following modifications. The paired non-fixed sub-samples of 1:10,000 longitudinal cultures were performed by removing a 400-µL sub-sample of the above described 30 mL experimental 1:10,000 longitudinal cultures at a time point 3 min after inoculation. These 400 µL sub-samples were added to two wells of a 96-well deep-well plate. The 96-well plate was immediately placed into the LifeScale with temperature-controlled plate holder set to either 25°C (*V. cyclitrophicus*) or 37°C (*E. coli*) and automatically sampled for measurement as described in “Single-cell mass and cell concentration measurements” section.

The paired fixed sub-samples of the 1:10,000 longitudinal 30 mL cultures in undefined, rich media (Fig. 4A and B, black filled symbols the same data as Fig. 1 and 2) were taken according to the fixation protocol in “Single-cell mass and cell concentration measurements” section.

The cross-sectional steady-state cultures for comparisons of fixed and nonfixed samples in all media were performed on different days than the longitudinal experiment cultures. The undefined, rich media cross-sectional experiments followed the same cultivation protocol as the 1:10,000 longitudinal cultures with three replicate cultures per strain and medium, but were further sub-cultured with a 1:10 dilution into pre-warmed media before they reached an OD_600_ of 0.1. Fixed and non-fixed cross-sectional samples were removed prior to these cultures (cumulative dilution 1:100,000) reaching an OD_600_ of 0.1. The defined, rich media cross-sectional experiments followed a modified version of the above cultivation protocol for 1:10,000 longitudinal cultures: on day 2, all cultures were diluted 1:200 instead of 1:100, and at an OD between 0.1 and 0.2 all cultures were further sub-cultured with a 1:1,000 dilution into pre-warmed media. The MOPS EZ rich + succinate cross-sectional fixation experiments were performed in these 1:1,000 cultures (cumulative dilution 1:200,000) before they reached OD_600_ 0.1. The MOPS EZ rich + glucose experiments were sub-cultured with a further 1:10 dilution into pre-warmed medium when they reached OD_600_ 0.1, then the cross-sectional fixation experiments were performed before these cultures (cumulative dilution 1:2,000,000) reached OD_600_ 0.1. Fixation details are below as described in “Single-cell mass and cell concentration measurements” section.

### Optical density measurements

Optical density at 600 nm (OD_600_) was measured on a Genesys40 spectrophotometer (Thermo Fisher) relative to uninoculated growth medium blanks in either cuvettes or test tubes where appropriate. The accuracy limits of the spectrophotometer (0.01–0.79 OD_600_) were determined with a dilution series of a stationary phase culture of *V. cyclitrophicus* 1G07 which was washed and re-suspended in a buffer that did not contain the macronutrients necessary for growth. OD_600_ of cultures was measured in the side-arm of the cultivation flask until approaching an OD_600_ value around 0.5, after which an additional 100 µL of sample was destructively removed from the flask to dilute and measure the true value within the accuracy limit of the spectrophotometer. Briefly, these samples were diluted 1:10 with fresh medium (0.1 mL sample into 0.9 mL medium) in a cuvette and immediately measured in the same spectrophotometer with a cuvette adapter. Diluted sample OD_600_ values were then multiplied by the dilution factor.

### Single-cell mass and cell concentration measurements

Several approaches for measuring single-cell mass have been developed in recent years, and they each have different properties which depend on their underlying measurement principle (37). The commercially available instrument we used in this study (LifeScale-Research Instrument, Affinity Biosensors) uses a vibrating cantilever with an internal microfluidic channel similar to previously described and published devices (38). As cells pass over the cantilever, they cause a shift in its resonant frequency, which is measured electronically at sub-millisecond resolution. This instrument allows for a high sample throughput which we required in our examination of the mass dynamics of batch cultures of different species at high temporal resolution in multiple growth media for both fixed and living cells. Cells transit the cantilever within about 8 ms at flow speeds used in these experiments, typically leading to about 40 separate frequency measurements per cell. The magnitude of the maximum frequency shift is proportional to the buoyant mass of the particle. Buoyant mass is the product of a cell’s volume and the density difference between cell material and its surrounding fluid. Changes in cellular buoyant mass can be directly translated to changes in cellular dry mass using dry density, which is the density of a cell’s dry material (17).

The in-built software automatically detects each particle based on the frequency data and user-specified settings (flow rate and sensitivity), then converts the frequency shift to buoyant mass in real time. The device was configured with automated sampling hardware and software and its flow speed was optimized for resolving particles at the smallest size possible (around 14 fg buoyant mass). The volume of fluid measured in each sample is also measured based on the transit time of individual cells passing over the sensor and the geometry of the fluidic system. This volume estimate, along with the total cell count, allows for accurate cell concentration estimates. We verified the calibration of the buoyant mass output of the experimental setup by measuring polystyrene beads of different sizes with NIST-certified diameter and density values Fig. S1.

All cultures were prepared with medium that was filtered with 0.1 µm pore size PES filters immediately prior to use to ensure it was free of any small particles that could interfere with cell measurements on the LifeScale cantilever. Negative controls of uninoculated medium were run on the LifeScale prior to inoculation to ensure the particle background of each medium batch was adequately low (concentrations of less than 1 × 10^5^ background particles/mL, comparable to ultrapure water controls).

Samples for fixation were removed from longitudinal batch cultures immediately after inoculation, and at approximately 30 min intervals for approximately 5–6 h. For 1:10,000 and 1:100 dilution longitudinal cultures, 11 fixation samples were taken over 5 h. For 1:1,000 dilution longitudinal cultures, 12–13 fixation samples were taken over 6 h.

For all cross-sectional steady-state fixation experiments, the fixed samples were taken out of the culture at a time designated *T*_0_ during steady-state and allowed to fix for various times (either 1 h, 1 day, 6 days, or 11 days) prior to measurement. The samples with different fixation times are treated as technical replicates to assess random variation due to sampling from the same population. Non-fixed samples were taken out of the steady-state cultures between 6 and 50 min before *T*_0_ and 4 and 45 min after *T*_0_ fixation samples as technical replicates to assess random variation due to sampling.

Sample fixation was performed by removing 1 mL of culture and adding it to pre-aliquoted microcentrifuge tubes containing 0.205 mL of ice-cold, 0.1 µm PES filtered formaldehyde (4% final concentration, from 23.5% methanol-free formaldehyde solution). Fixation samples were kept on ice for 1 h before storing permanently at 4°C. Measurements of longitudinal experiment fixation samples for single-cell mass and cell concentration were performed at least 1 day after samples were fixed. Measurements of cross-sectional experiment fixation samples for single-cell mass and cell concentration were performed between 1 h and 11 days after samples were fixed. The concentration of cells in successive time point samples of longitudinal experiments changed over time due to growth, but the LifeScale sensor can only operate accurately at concentrations between approximately 1 × 10^4^ and 3 × 10^7^ cells/mL. Therefore, we removed a small volume from each longitudinal experiment fixation sample after vortexing to re-suspend cells, and diluted it in 0.1 µm PES filtered growth medium (where necessary) to target a concentration of 1 × 10^7^ cells/mL. Measured concentrations were then adjusted for this sample dilution. We do note the 1:10,000 cultures in the longitudinal experiment were below the 1 × 10^7^ cells/mL target concentration until near the end of the experiment, so many samples from these cultures were counted without any dilution. This resulted in fewer cells counted per sample of the 1:10,000 cultures than the 1:100 cultures or the 1:1,000 cultures in the longitudinal experiment. Sample sizes (cells per sample) for all figures are included in Table S1. Sample sizes differ slightly for mass (Fig. 1 and 2A through F; Fig. 3A through D) and concentration (Fig. 1 and 2G through I; Fig. 3E through F) due to some particles passing through the mass sensor nearly simultaneously. If two particles pass through the sensor simultaneously and only one particle’s mass can be accurately measured, the software registers one particle for mass purposes (one measured particle) but two particles for concentration purposes (two detected particles). Therefore, the discrepancy in sample size for different figure panels measured from the same sample relates to the difference between measured and detected particles.

Assessing the effect of fixation required measuring non-fixed samples. The longitudinal experiments required a different approach for measuring non-fixed samples than that of the cross-sectional steady-state experiments. In the longitudinal fixation effect experiments, two replicate non-fixed samples were added into separate wells of a 96-well plate, and the plate was placed in the LifeScale and measured for 4 min per well (with a washing step between each replicate) in series, leading to an approximate cycle of 10 min to measure both replicates. This cycle was set to repeat 32 times for both species. *V. cyclitrophicus* samples reached 32 cycle measurements, but *E. coli* samples were measured 23 times before they went above the saturation point for accurate sensor operation set by the LifeScale software (3 × 10^7^ cells/mL). In the cross-sectional experiments, non-fixed samples of 50–100 µL were removed from the batch culture at two time points (before and after fixation), added into separate wells of a 96-well plate, and the plate was placed in the LifeScale and measured within 5 min at the appropriate temperature setting for each species (25°C for *V. cyclitrophicus* or 37°C for *E. coli*). Samples were measured for 4–5 min on the LifeScale.

### Data analysis and statistics

Cell concentration and buoyant mass data were exported from LifeScale software in .csv format. All further analysis, statistics, and figure generation were performed using custom Python scripts and the R statistical programming language using base R (39), the packages tidyr (40), ggridges (41), and lmerTest (42). Statistical analysis of steady-state fixed versus non-fixed samples was performed as an ANOVA mixed-effects model using log-transformed mass as a response variable and the predictor variables as follows: treatment as a fixed effect (two levels: fixed and non-fixed), sample as a random variable nested within treatment (levels for undefined, rich media panels A and B: fixed = 1 h, 1 day or 3 days, and 6 days or 11 days, non-fixed = before and after; levels for defined, rich media panels C and D: fixed = 1 day, non-fixed = before and after), and replicate as a random variable (levels for undefined, rich media panels A and B: rep 3, rep 4, and rep 5; levels for defined, rich media panels C and D: rep 5 and rep 6).

## Data Availability

Data used in this study and code necessary to re-generate all figures are available publicly on the Polz Lab github (https://github.com/polzlab).

## SUPPLEMENTAL MATERIAL

Fig S1 mbio.01585-23-s0001.eps LifeScale calibration verification and fluid density measurements.

Figure S2 mbio.01585-23-s0002.eps Mass variation among *V. cyclitrophicus* cells in MB2216 batch cultures.

Figure S3 mbio.01585-23-s0003.eps Mass variation among *E. coli* cells in LB batch cultures.

Figure S4 mbio.01585-23-s0004.eps Mass variation among *E. coli* cells in MOPS EZ rich batch cultures with two different primary carbon sources.

Table S1 mbio.01585-23-s0005.xlsx Sample size and metadata for figures.

ASM does not own the copyrights to Supplemental Material that may be linked to, or accessed through, an article. The authors have granted ASM a non-exclusive, world-wide license to publish the Supplemental Material files. Please contact the corresponding author directly for reuse.
